# Estimating the genetic merit of sires by using pooled DNA from progeny of undetermined pedigree

**DOI:** 10.1186/s12711-017-0303-8

**Published:** 2017-02-28

**Authors:** Amy M. Bell, John M. Henshall, Laercio R. Porto-Neto, Sonja Dominik, Russell McCulloch, James Kijas, Sigrid A. Lehnert

**Affiliations:** 1 CSIRO Agriculture, F D McMaster Laboratory Chiswick, Armidale, NSW 2350 Australia; 2 CSIRO AgricultureQueensland Bioscience Precinct, Brisbane, QLD 4067 Australia

## Abstract

**Background:**

DNA-based predictions for hard-to-measure production traits hold great promise for selective breeding programs. DNA pooling might provide a cheap genomic approach to use phenotype data from commercial flocks which are commonly group-mated with parentage unknown. This study on sheep explores if genomic breeding values for stud sires can be estimated from genomic relationships that were obtained from pooled DNA in combination with phenotypes from commercial progeny.

**Methods:**

Phenotypes used in this study were categorical data. Blood was pooled strategically aiming at even pool sizes and within sex and phenotype category. A hybrid genomic relationship matrix was constructed relating pools to sires. This matrix was used to determine the contribution of sires to each of the pools and therefore phenotype category by using a simple regression approach. Genomic breeding values were also estimated using the hybrid genomic relationship matrix.

**Results:**

We demonstrated that, using pooled DNA, the genetic performance of sires can be illustrated as their contribution to phenotype categories and can be expressed as a regression coefficient. Genomic estimated breeding values for sires were equivalent to the regression coefficients and are a commonly used industry tool.

**Conclusions:**

Genotyping of DNA from pooled biological samples offers a cheap method to link phenotypic information from commercial production animals to the breeding population and can be turned into information on the genetic value of stud sires for traits that cannot be measured in the stud environment.

## Background

Genomic predictions have had a significant impact on livestock breeding systems for which large reference populations with genotypic and phenotypic information exist [1]. The opportunity to develop genomic predictions has been addressed by forming specialized nucleus herds or flocks that are extensively phenotyped for the beef and sheep industries [2, 3]. Commercial flocks provide an unmined resource of abundant phenotypes that are assessed or measured during routine commercial husbandry procedures. Using commercial phenotypes for genetic evaluation has been hindered by the fact that performance records are not usually captured, animals are often not individually identified and/or no parentage information exists because flocks or herds are group-mated. However, in combination with affordable genotyping, commercial phenotypes could add genetic information on sire performance under commercial conditions. In spite of the decreasing cost of genotyping, it would still be a substantial expense to assess the performance of sires in a commercial environment, based on individual genotypes. Commercial phenotypes can be exploited in a cost-effective manner without needing to capture individual records by strategically pooling DNA and using it in a genetic evaluation approach [4].

Allele frequencies can be estimated from pooled genotype data and subsequently estimated effects of single nucleotide polymorphisms (SNPs) from a genome-wide association study (GWAS) have been demonstrated to be equivalent to effects of SNPs from individual genotyping [4, 5]. This approach reduces the cost of GWAS [6–8]. It has also been demonstrated that the genetic merit of sires can be estimated from pooled DNA in combination with phenotype information collected on commercial properties during routine husbandry procedures [5, 9].

The objective of this study was to determine whether, in the absence of individual genotypes, pooled DNA genotypes can be used in combination with commercial progeny records on a categorical phenotype to estimate genomic breeding values and to illustrate these in a regression approach as a sire’s genetic contribution to a particular phenotype. This approach could be a cost-effective commercial progeny test to inform stud breeders and commercial producers on the genetic suitability of sires for specific environments.

## Methods

### Animals

Approval was granted (AEC approval number 1582) to use the animals in this study by the Animal Ethics Committee of the Australian Animal Health Laboratory, Victoria. Two thousand six hundred 13 to 14 months old Merino sheep were available for the trial. The sires of the sheep in the study were obtained from a stud in southern New South Wales which had been the sole provider of rams to the commercial property for many years. For management purposes, the sheep were maintained in two groups according to sex, ewes (female) and wethers (castrated males).

The phenotype of interest in this study is called “dag score” and describes the amount of faecal soiling in the breech area of the sheep. Breech soiling devalues the wool harvested from the sheep, increases time and management costs of routine husbandry practices and can predispose the animal to increased risk of flystrike. Dag score is a heritable trait with a heritability of about 0.35, depending on the age of the animal at the time of phenotype assessment [10], but is not expressed in all environments. Dags were a problem for this specific commercial property, but the stud from which this commercial property sourced rams was located in an environment where dags do not occur and no information existed for the sires’ propensity for dag formation. The dag score phenotype was assessed on a scale of 1 (no soiling) to 5 (heavy soiling) based on Visual Sheep Scores, a commercial guide for visually assessed traits developed by Australian Wool Innovation Limited (AWI) and Meat and Livestock Australia (MLA) [11]. Dag score is a visual assessment and a large number of animals can be phenotyped in a short period of time. All sheep were moved through the sampling race for scoring and bleeding until a maximum of 80 individuals per sex and dag score was reached, resulting in a subset of 400 males and 386 females as outlined in Table 1.

**Table 1:** Description of phenotypic values for dag scores [11] and pool sizes (number of pools × number of individuals in the pool)

Dag score	Description	Male pools	Female pools
1	No dags	2 × 40	2 × 40
2	Some dags around the breech area	2 × 40	2 × 40
3	Dags around the breech area, some dags reaching down the inner hind leg to above the hock	2 × 40	2 × 40
4	Dags around the breech area, reaching down the inner hind legs to the hock and extend out	2 × 40	2 × 40
5	Extensive dags around breech and extending down the hind legs to the pasterns, covering extensive amount of the hind legs	2 × 40	2 × 33

### Pooling design

Pool formation within contemporary groups or fixed effects has been suggested as the most effective pooling design [12]. Generally no fixed effects or contemporary group information is recorded on commercial properties. For this property, sex was the only known fixed effect. Within the two sexes and five dag scores, with 80 individuals each, samples were randomly split into two replicates, with 40 individuals per pool, with the exception of the pools of females with dag score 5, for which only 66 samples were available, and which formed two pools of 33 samples each. This resulted in a total of 20 pools. Although parentage of individual sheep was unknown, 33 of the 45 sires used to produce the flock of sheep sampled were still present for DNA sample collection.

Once samples were assigned to pools, samples of 20 μL of whole blood from each individual sample within each pool were combined to create a pooled blood sample. 200 μL of the pooled blood was then used for genomic DNA extraction following manufacturer’s instructions (Qiagen DNeasy^®^ Blood and Tissue Kit).

For genotyping, each of the pooled DNA samples and the 33 individual sires were assayed with the Illumina Ovine SNP 50 chip [13]. To obtain the relevant data, the Illumina GenomeStudio™ software was formatted to export a variable called “B-allele frequency” [14, 15]. These are quantitative estimates of the proportion of the alternative SNP allele. For an individual genotype, the B-allele frequency is 0 or 1 for homozygous individuals and 0.5 for heterozygous individuals. For a pooled sample, the B-allele frequency for a SNP can range from 0 to 1. Genotypes for pooled DNA were generated based on the diploid clustering algorithm [14]. Pooled DNA presents like polyploidy data, which consequently, renders some of the routine quality control parameters, such as GenCall and HetExcess [15], which are not meaningful since they aim to eliminate rare alleles, whereas they are informative for this study. Other classic quality control steps such as thresholds for minor allele frequencies are not applicable either. The aim of the approach presented here is to relate allele frequencies from the pooled progeny data to the individual sire data and rare alleles provide useful information to this process.

### Statistical analysis

#### Hybrid genomic relationship matrix based on pooled DNA

Sire relationships with pools were estimated through a genomic relationship matrix. The method described here has previously been termed “hybrid genomic relationship matrix” (h-GRM) because it consists of three blocks of relationships, i.e. (1) between pools, (2) between individual sires, and (3) between pools and sires [5]. The first method of VanRaden was applied [16], but instead of discrete genotype calls, B-allele frequencies were used which describe the allele frequency of the second allele at each locus.

VanRaden uses matrix ${\mathbf{M}}$ which specifies which SNP alleles each individual inherited, and the dimensions of ${\mathbf{M}}$ are the number of individuals (n) by the number of loci (i), containing values −1 and 1 for individuals that are homozygous for the respective allele of the locus and 0 for heterozygous individuals. For this study, matrix ${\mathbf{M}}^{*}$ was used, which is equivalent to ${\mathbf{M}}$. Let ${\mathbf{M}}^{*} = \left( {{\mathbf{Bfreq}} - 0.5} \right) *2$, with ${\mathbf{Bfreq}}$ being a matrix of the dimension of number of sires (*s*) plus the number of pools (*p*) by the number of loci (*i*), containing B-allele frequencies. The B-allele frequencies for sires were 0.0, 0.5, and 1.0 for the homozygote, heterozygote and other homozygote, respectively, and for pools these were expressed as a real number between 0 and 1. For vector ${\mathbf{P}}^{*}$ of size (1 × (*s* + *p*)), the same formula is used as for ${\mathbf{P}}$ by VanRaden, but ${\mathbf{P}}$ is of size I [16]. Let vector ${\mathbf{P}}^{*}$ be ${\mathbf{P}}^{*} = \left( {{\mathbf{freq}} - 0.5} \right) *2$ with ${\mathbf{freq}}$ being a vector of B-allele frequencies for sires and pools.

Let matrix ${\mathbf{Z}}^{*}$ be the difference between ${\mathbf{M}}^{*}$ and ${\mathbf{P}}^{*}$, equivalent to ${\mathbf{Z}} = {\mathbf{M}} - {\mathbf{P}}$ [16].

Matrix ${\mathbf{G}}^{*}$ was calculated in the same way as ${\mathbf{G}}$ by VanRaden with ${\text{G}}^{ *} = \frac{{{\text{Z}}^{ *} {\text{Z}}^{ * '} }}{{2\sum p_{i} \left( {1 - p_{i} } \right)}}$, with *p*_*i*_ the frequency at locus *i* [16]. For this study, we re-scaled the B-allele frequencies as described above for ${\mathbf{P}}^{*}$ to obtain *p*, but with missing values set to 0.5.

Then, the distribution of pool phenotypes is not necessarily a scaled equivalent of the distribution of phenotypes in the population. In our sheep data, pool sizes were fairly even. Although not necessary in this case, matrix ${\mathbf{H}}^{*}$ was used to scale h-GRM elements to account for potential differences in pool sizes. Therefore, to account for differences in pool sizes, we scaled the elements of ${\mathbf{G}}^{*}$ by the square roots of the product of the diagonals to produce a matrix ${\mathbf{H}}$ with elements ${\text{H}}_{ij} = \frac{{{\text{G}}_{ij}^{ *} }}{{\sqrt {{\text{G}}_{ii}^{ *} \times {\text{G}}_{ij}^{ *} } }}$. This is similar to multiplying the relationships by the number of individuals (n) in the pool. The problem is that n is not known with certainty, because the “effective n” depends on the variations in the concentration of DNA in the blood samples and on the accuracy of mixing the samples. Since this is impossible to determine, the scaling of ${\mathbf{G}}^{*}$ into ${\mathbf{H}}$ provides an analytical solution to the technical problem.

### Regression analysis of sire relationship with pool on dag score

The relationship of the sires with pools was regressed on the dag score. The DNA pooling strategy was based on phenotype category, therefore, the linear relationship between genetic contribution of a sire and phenotype categories is expressed in the regression coefficient. In this study, a positive regression slope indicates a higher degree of relatedness of the sire to the pools with higher dag scores. Conversely, a negative slope indicates higher relatedness of the sire to the pools with low dag score. Therefore, the latter would characterize sires that are genetically favorable for dag score.

For the regression approach, we fitted the following model:

$${\mathbf{Y}}_{\text{i}} =\upbeta_{0} +\upbeta_{1} {\mathbf{X}}_{{{\text{i}}1}} +\upbeta_{2} {\mathbf{X}}_{{{\text{i}}2}} +\upvarepsilon_{\text{i}} ,$$

where ${\mathbf{Y}}_{\text{i}}$ is the vector of the GRM relationships of sire i with the pools, β_0_ is the intercept, β_1_ and β_2_ are regression coefficients, ${\mathbf{X}}_{{{\text{i}}1}}$ is a vector of the fixed effect of sex of contributors to the pools, to account for any stratification due to sex, ${\mathbf{X}}_{{{\text{i}}2}}$ is a vector of the fixed effect of dag phenotype of contributors to the pools, and ε_i_ is a random error term.

*P* values of the regression coefficient were empirically validated through permutation testing. Sires’ phenotype data were permuted 100 times within sex. The distribution of counts for the real data compared to the distribution of counts for the permuted data using a Pearson’s Chi squared test with simulation was used to derive the significance level. Results were tabulated, and means were calculated for each sire’s genomic relationship to a pool and for the slope of the relationship between each sire and each pool calculated.

### Genomic breeding values from pooled data

Genomic breeding values were estimated using h-GRM in a genomic best linear unbiased prediction (gBLUP) approach in ASReml [17]. The following model was fitted:

$${\mathbf{y}} = {\mathbf{Xb}} + {\mathbf{Zg}} + {\mathbf{e}},$$

where ${\mathbf{y}}$ is a vector of dag phenotypes for pools and missing values for sires, ${\mathbf{X}}$ is a design matrix relating the fixed effect of sex to each pool and sire, ${\mathbf{b}}$ is a vector of sex for each pool and sire, ${\mathbf{Z}}$ is a design matrix allocating records to genetic values to each pool and sire, ${\mathbf{g}}$ is a vector of genetic effects for each pool and sire, ${\mathbf{e}}$ is a vector of random normal deviates with variance $\upsigma_{\text{e}}^{2}$.

It was assumed that ${\text{var}}\left( {\text{g}} \right) = {\mathbf{G}} *\upsigma_{\text{g}}^{2}$, where g is the h-GRM and $\upsigma_{\text{g}}^{2}$ is the genetic variance in this model. As outlined above, the gBLUP model fits the dag phenotype of the pool as the dependent variable and includes sex as the only known fixed effect. The previously constructed h-GRM was used in the model instead of the numerator relationship matrix.

## Results and discussion

### Pooling strategy

An appropriate pooling strategy strikes a balance between cost effectiveness and accuracy. Here, we were not in a position to assess our pooling strategy on accuracy, but previous studies suggested that 64 pools of 46 individuals each estimated SNP effects equally well as individual genotypes [12]. We used pools of 33 to 40 individuals each. Although cost and accuracy are considerations, the numbers and sizes of pools are mainly determined by the total number of animals, phenotype distribution and contemporary group structure. Here, pools were formed within fixed effects, as suggested by [12], with sex being the only known fixed effect. Pooling strategies based on contemporary group information and phenotype are likely to have uneven contributions of sires to each of the pools, in particular if it is a moderately heritable trait, such as dag score in this study. Since a larger number of smaller pools would estimate allele frequencies more accurately [12], in this study, groups of animals of the same sex and phenotype category were randomly split into smaller pools of 40 because this was demonstrated to be an appropriate pool size [12]. In a study on beef cattle, 15 to 28 individuals were pooled, but again, the number of pools and resulting pool sizes were determined by the contemporary group structure and phenotype categories [5]. More comprehensive knowledge on contemporary group effects, e.g. year of birth, might have added more objective information to the pooling strategy, but in a commercial setting, individual records are not kept and ear tags only indicate year of birth. Therefore, pooling strategies for commercial data might often have to take a pragmatic approach. However, one of the attractive characteristics of a pooling approach is the ease of the logistics of phenotype collection, which should slot into commercial husbandry procedures without major additional workload for the producer.

### Genomic relationships

**Fig. 1:**
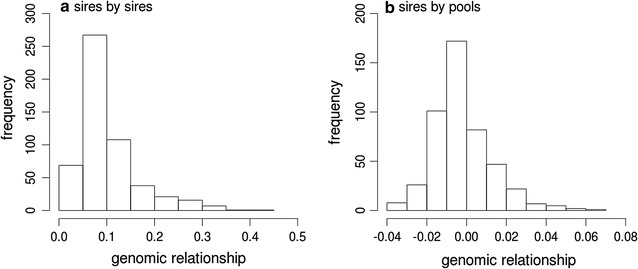
Frequencies of the genomic relationships between **a** sires and other sires within the study and **b** sires and the pools within the study

The interpretation of the genomic relationships between sires provides some insights into the choice of sires for the commercial flock. Genomic relationships between sires, which are the off-diagonal values of the sire-by-sire block of the h-GRM, ranged between 0 and 0.5, with the majority around 0.1 (Fig. 1a). The commercial property is a pure-bred, self-replacing Merino flock where the replacement rams are sourced from one stud and have been for a number of years. The distribution of genomic relationships suggests that the sires of the commercial property, in spite of being from the same stud, had a low degree of relatedness. Only a small number of sires were highly related.

As a consequence of the low sire-by-sire relationships, we would expect the relationships between sires and animals that contributed to a pool and which are not progeny of the sire to be also low. Multiple sires contribute to a pool, therefore the sire-by-pool relationships can be expected to reflect a wider range of expected values than the sire-by-sire relationships. For a heritable trait, this may be enhanced through the pooling by phenotype strategy, if more related sires contribute more often to pools with the same phenotype. The frequencies of the genomic relationship estimates between sires and pools are plotted in Fig. 1b. The majority of the estimates ranged from −0.02 to 0.02. Within breed one might expect to see only positive relationship values, but since the relationships are estimated based on identity-by-state, the range of relationship values depends on the population of genotypes that are being used. Therefore, it is not unusual to see negative relationships for the lower relationship values between sires and pools. However, the variation in the genomic relationships between sires and pools indicates that sire contributions to pools vary and it was demonstrated that this variation can be exploited to obtain information on the genetic merit of sires.

### Sire genetic merit from pooled DNA

To determine whether the relationships between sires and pools were associated with the phenotypes of the pools, for each sire we regressed the genomic relationships on the pool dag scores. Departure from the null hypothesis, assuming no association between sire-pool relationship and pool phenotype, was assessed by examining the distribution of the significance levels of the regressions. The regressions of sire relationship on dag score were more often significant than would be expected by chance. For example, 10 of 33 sires (30%) were significant at the 5% level (Table 2), far more than would be expected by chance, and this trend was also observed at other significance levels. To check that this result was not due to our small sample size, we performed the analyses again after permuting the pool phenotypes. When *P* values were allocated to significance level classes and the counts compared for the observed and permuted data, the Chi square test was significant (*P* = 0.015), which indicates that the variation observed between sires in the contributions to pools of different dag scores is likely to be real, as one would expect for a heritable trait.

**Table 2:** Proportions of analyses that were significant at various levels for the observed data and for the data with phenotypes permuted

	Significance level				
	0.05	0.01	0.005	0.001	0.0005
Observed	0.300	0.120	0.061	0.030*	0.030*
Permuted	0.049	0.011	0.005	0.001	0.001

* 0.0303 is one out of 33 sires

**Fig. 2:**
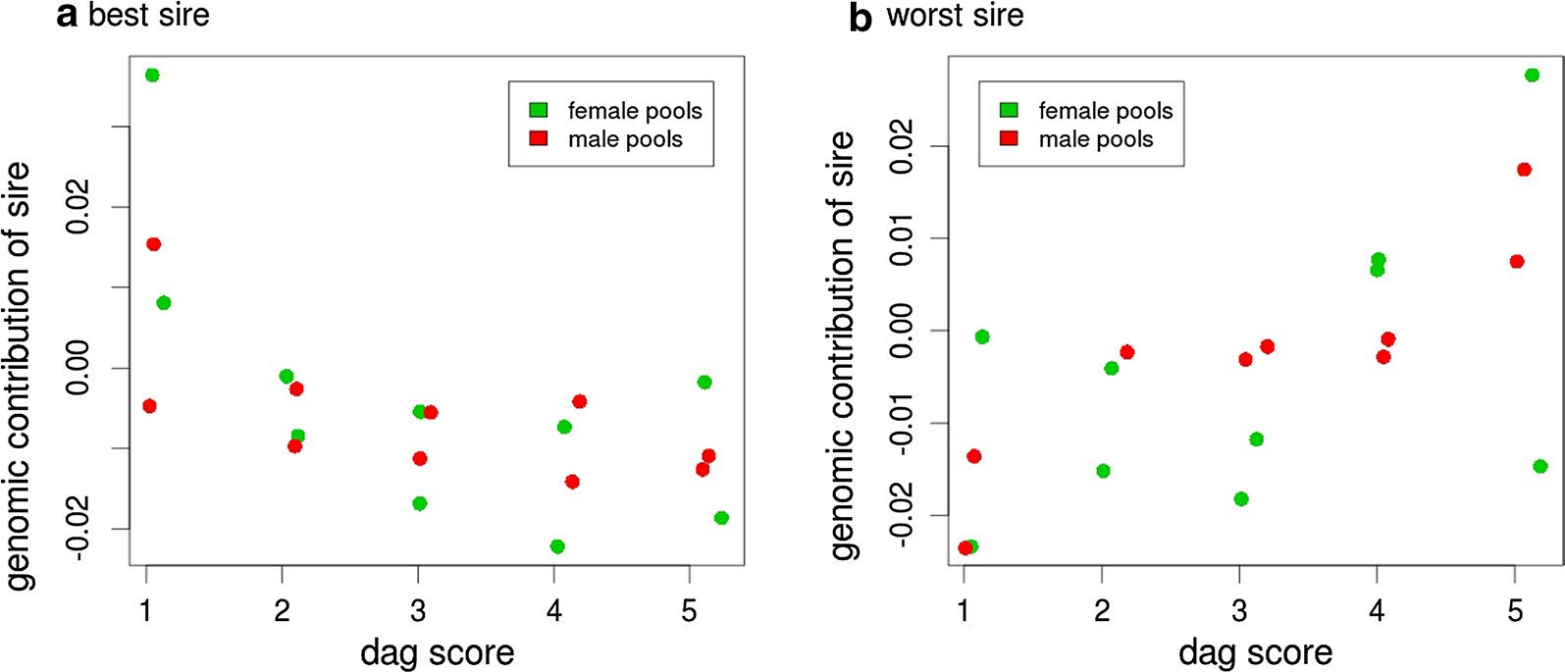
An example of the best versus the worst sire when a sire’s genomic contribution is regressed on the dag score phenotype. The best sire has the most negative slope, indicating a greater contribution to lower dag score phenotypes. The worst sire has the more positive slope, contributing more to higher dag score phenotypes

The “best” and “worst” sires with respect to the regression coefficients for dag score are illustrated in Fig. 2a, b. The best sire had the strongest relationship with lower dag score phenotypes, and therefore the most significant negative slope of the regression line (Fig. 2a). The worst sire had the most significant positive slope of the regression line and therefore a stronger relationship with the higher dag score phenotype. This suggests that the slope of the regression can be used to obtain information on genetic sire performance for the categorical phenotype of dag score.

The replicate pools for each phenotype category, i.e. two male and two female pools (Fig. 2) show that a random split of pools influenced the relationships of pools with sires. Figure 2 demonstrates that the random split resulted in the progeny from the same sire to be divided unevenly across the replicate pools. For example, the relationships of the replicates of female pools for the best and worst sire are in many cases different. As discussed earlier, more objective information on the formation of appropriate size pools might assist in devising a strategy that does not decrease the number of progeny of a sire to a particular pool.

**Fig. 3:**
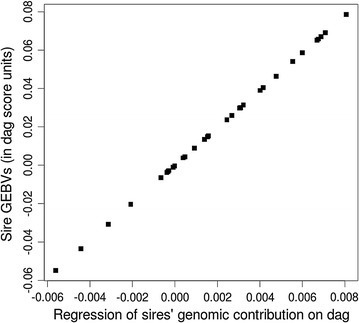
Relationship between sires’ genomic estimated breeding value (in dag score units) and the coefficient of the regression from sires’ genomic contribution on dag score

The equivalence of the regression approach compared to genomic estimated breeding values (GEBV) obtained with a gBLUP approach is shown in Fig. 3. Not only did we demonstrate that the contribution of a sire to a phenotype category can be estimated in a regression approach, but this can be translated into a breeding value as it is commonly known and used in the stud industry.

### Implications

A probability-based approach was suggested to estimate a numerator relationship matrix for multiple sire combinations [18], but genomic information provides not only a more rigorous approach to identify parentage of an individual but also the representation of a sire’s genes to a pool of individuals [9]. There are likely to be tradeoffs in accuracy in genomic breeding values that are derived from pooling compared to individual genotypes, which could not be demonstrated from the data in this study. However, assaying pooled DNA is cheap compared to individual genotypes and the resulting genomic breeding values provide a ranking for commercial performance of the sires under evaluation, which fills a gap for which there was previously no available information.

In the hierarchical structure of most livestock industries, where genetic improvement is undertaken in the stud breeding sector, and the genetic gains flow to the commercial sector through the sale of sires and semen for artificial insemination, no performance information from the commercial sector returns to the stud. This is a lost opportunity to genetically characterize sires across environments. The approach demonstrated in this study has multiple benefits for the stud and commercial sector. It provides an opportunity to apply cost-effective genomic technologies to harvest commercial performance data, which is either routinely measured or assessed during general husbandry procedures or could be obtained with only small additional labour investment. The resulting information on the sires provides the commercial producer with the knowledge of which sires have contributed to particular phenotypes, which can inform future sire or stud selection. Although, in this study sires were evaluated retrospectively, individual genotypes of related current generation sires could be included in a pooling study to obtain information of their genetic merit in a commercial environment [5].

For studs, the proposed pooling approach offers genomic breeding values for their sires for traits that are impossible to obtain in the stud environment, e.g. disease or performance in a commercial environment. It has been demonstrated that significant genotype × environment (G × E) interactions exist in the Merino industry between the stud and commercial level [19] and the information generated from the DNA pooling approach could inform such G × E interactions.

The next step in defining the value proposition of DNA pooling for the livestock industries is to establish the loss in accuracy of pooling versus individual genotyping. Here, we demonstrated that the contribution of sires to phenotypes reflects their genetic merit and it can be translated into a breeding value which is a known industry tool. DNA pooling is a cheap approach that can inform a knowledge gap on commercial performance of sires which can be exploited by stud breeders and producers.

## Conclusion

Estimates of the genetic merit of sires using pooled DNA from progeny in a commercial production system can be determined using this technique, and it can provide a cost effective option to inform on the performance of sires for traits that cannot be measured in the stud environment, but are important for commercial operations, and to ultimately increase the amount of data available to stud breeders to inform on the genetic value of sires for a range of traits.
